# A Case of Puerperal Convulsions

**Published:** 1867

**Authors:** J. Ramsay Flood

**Affiliations:** Detroit, Mich.


*A Case of Puerperal Convulsions.—Additional testimony in favor of Belladonna.* By J. RAMSAY FLOOD, M.D., of Detroit, Mich.

On the night of the 30th of January last, I was called to see Mrs. B., who was suffering from puerperal convulsions. When I reached her, she was in the fourth spasm. After the paroxysm had subsided and consciousness returned, patient complained bitterly of pain in her right shoulder, and, upon examination, an anterior luxation of the right arm at the shoulder joint was discovered. This was produced by the second paroxysm, inasmuch as patient had not complained previous to that time. The lady had been unavoidably and unexpectedly detained on one of the river-boats for some four hours, and what is strange at this season, without fire or any means of keeping warm. Her whole system was thoroughly chilled, and when she reached home complained of lancinating pains through her chest and shoulders. The following morning she was seized with violent convulsions. From the friend's data, she had not gone to full term; but they were doubtless in error, as the child bore the appearance of full development. Patient being of full habit, by the aid of venesection to the extent of ℥viij of blood and antispasmodics, the convulsions were, in some degree, controlled—their intensity much lessened. Uterine contractions were very feeble from first to last—too feeble to produce any effect in dilating the os, nor did large and oft-repeated doses of the fluid extract of ergot augment its contractile force. The drug was pure and of officinal strength. After waiting some twelve hours in anxious expectation for some effect from the administered drug; it was evident I could expect no aid from that source, and made my first use of the solid extract of Belladonna as a dilator of the os uteri. The application was freely made to the os by the aid of the speculum, and I was not a little surprised at the result. In thirty minutes, the os, which had not been more than half an inch in diameter for at least twelve hours, was now fully three inches in diameter, and, in forty-five minutes, the head passed the os and became engaged in the inferior strait; nor had the uterine contractions been any more vigorous than previous to the application of the Belladonna. Having heard many physicians express a disappointment in the use of this drug, having seldom or never obtained the effect which they were induced to expect from the books, I regarded its use in this case as an experiment, and consequently watched its effect with more than ordinary vigilance, and regard it as a fair test of the action of the pure drug upon rigidity of the os in the gravid uterus. And having thus been so happily disappointed, I feel it a duty to bear additional testimony to my brothers of the profession of its efficacy as a dilator of the os uteri.

The convulsions continued, but, as stated, with much less severity, (numbering in all twenty-one) and ceased immediately upon delivery. The child, as is usual under such circumstances, was still-born; delivery being performed by the aid of forceps, owing to inertia of the womb.

Mrs. B. assumed full charge of her household duties twelve days after her confinement, notwithstanding my admonitions to the contrary, and is now in the enjoyment of good health.

